# Immunogenicity of a Fusion Protein Comprising Coli Surface Antigen 3 and Labile B Subunit of Enterotoxigenic *Escherichia coli*

**DOI:** 10.6091/ibj.1344.2014

**Published:** 2014-10

**Authors:** Masoome Alerasol, Seyed Latif Mousavi Gargari, Shahram Nazarian, Samane Bagheri

**Affiliations:** 1 *Dept. of Biology, Shahed University, Tehran, Iran;*; 2 * Depent of Biology, Imam Hussein University, Tehran, Iran*

**Keywords:** Recombinant vaccine, Enterotoxigenic *Escherichia coli* (ETEC), *cstH*, *eltB*

## Abstract

**Background:** Enterotoxigenic *Escherichia coli *(ETEC) strains are the major causes of diarrheal disease in humans and animals. Colonization factors and enterotoxins are the major virulence factors in ETEC pathogenesis. For the broad-spectrum protection against ETEC, one could focus on colonization factors and non-toxic heat labile as a vaccine candidate. **Methods:** A fusion protein is composed of a major fimbrial subunit of coli surface antigen 3, and the heat-labile B subunit (LTB) was constructed as a chimeric immunogen. For optimum level expression of protein, the gene was synthesized with codon bias of *E. coli*. Also, recombinant protein was expressed in *E. coli* BL21DE3. ELISA and Western tests were carried out for determination of antigen and specificity of antibody raised against recombinant protein in animals. The anti-toxicity and anti-adherence properties of the immune sera against ETEC were also evaluated. **Results:** Immunological analyses showed the production of high titer of specific antibody in immunized mice. The built-in LTB retains native toxin properties which were approved by GM_1_ binding assay. Pre-treatment of the ETEC cells with anti-sera significantly decreased their adhesion to Caco-2 cells. **Conclusion:** The results indicated the efficacy of the recombinant chimeric protein as an effective immunogen inducing strong humoral response. The designated chimer would be an interesting prototype for a vaccine and worthy of further investigation.

## INTRODUCTION

Enterotoxigenic *Escherichia coli *(ETEC) is mostly prevalent in developing countries, and it causes diarrhea among children [1, 2] and visitors [1-3]. Diarrhea, which is caused by ETEC infection, leads to symptoms ranging from a nearly mild indisposition to a dehydrating condition resembling cholera disease [3]. In acute conditions [4], the ratio of death among children under five years old is 400,000 annually [2]. Colonization factor antigens or adhesions [5] and enterotoxins (heat stable and heat labile) [6] are two major ETEC virulence factors. Colonization factors are categorized as colonization factor antigens (CFA) or coli surface antigens (CS) [5]. The study of human ETEC strains revealed diverse antigenic sorts of CFA [3, 7]. CFA/I, CFA/II, and CFA/IV are predominant ones isolated from 50 to 75% of individuals infected with ETEC strains in different geographic areas throughout the world [8]. CS1, CS2, and CS3 are subsets of CFA/II and CFA/IV composed of CS4, CS5, and CS6 antigens [1, 9]. CFA/I and CS1-CS6 have higher prevalence among a variety of (>25) identified colonization factors [8]. CS3 is the most stated serotype protein in CFA/II family [6]. It is expressed alone and/or accompanied by CS1 and CS2. CS3 is characterized as a form of a flexible, fine, wiry thread with approximately 2 nm in diameter [10]. CS3 gene clusters are made up of *cstA *to –*H* genes, where *CstH* encodes the major fimbrial subunit of a 17.5-kDa precursor. 

Two categories of toxins, heat stable and heat labile, are produced by ETEC [11]. Heat stable is a small molecule with poor ability to stimulate immunogenic reaction [7]. Heat labile has been proved as an immunogen in human [8] that stimulates mucosal and systemic immune responses [11]. It consists of two subunits, known as A and B, with B subunit (labile B subunit [LTB]) devoid of toxicity [11, 12], and it is in charge of toxin binding process [11, 13].

Even though the main treatment for ETEC diarrheal illness has been antibiotic therapy, the numbers of available antibiotics have been limited by increasing in anti-microbial resistance [14]. One of the most important prevention methods against ETEC is vaccine development. Although O antigens stimulate antibody responses, their diversification are too high to be used as vaccine [2]. Besides, flagellar and lipopoly-saccharide serogroups variation causes prohibition of the O and H antigens to be objective points in vaccine design [15]. Thus, the majority of vaccine development strategies depend on multivalent approaches, containing colonization factors with a heat-labile portion that provide a vast extent inclusion [8, 16].

Factors such as colonization and heat sensitivity of protective antigens imply that a fusion vaccine consisting of a heat-labile toxoid and CS3, CFA/I, and CS6 would cover more than 85% of ETEC isoltes worldwide. Existence of more widespread CFA in a vaccine formulation affords an encouraging vaccine [2, 10, 14] integrity, of which would be enhanced by adding anti-heat-labile immunity [5].

Until now, a lot of works have been carried out to generate ETEC vaccines, and all have considered heat labile and/or the colonization factors. Injection of purified heat labile and fimbriae in transcutaneous form, oral administration of microencapsulated purified fimbriae, DNA vaccines, killed whole and live attenuated ETEC cells, and expression of heat-labile B by transgenic plants are attempts made in this field [17, 18]. Adhesion-toxin chimeric antigens have the ability to induce anti-toxin and anti-adhesion immunity simultaneously [13] and such a vaccine deserves to be ideal because of conferring protective immunity against ETEC virulence factors [5]. In the present research, we designed a chimeric vaccine containing B subunit of heat labile and the major subunit of CS3 (*cstH*) to cover immunity against both ETEC virulence factors. 

## MATERIALS AND METHODS

***Bacterial strains, plasmids, and media.*** *E*. *coli *strains TOP10 and BL21DE3 (Invitrogen, Carlsbad, CA) were used to construct recombinant strains. Plasmids pET28a and pET32a (Novagen, USA) were served as vectors for expression of recombinant genes. The constructed *E*. *coli *strains were grown in Luria Bertani (LB) medium containing kanamycin (70 μg/ml) and ampicillin (50 μg/ml) (Merck, Germany) at 37°C for protein expression. Stock cultures (positive colonies) were maintained in 20% glycerol at -70°C.

***Construction and cloning of cs3 (cstH-eltB):*** A bioinformatic analysis was carried out to design and optimize the sequence with *E. coli* codon usage [19]. A suitable linker (EAAAK)4 was incorporated between the *eltB* and *cstH *gene sequences. I-TASSER software was used to estimate tertiary structure prediction. The chimeric encoding gene was synthesized by Shine Gene Molecular Biotech, Inc. (Shanghai, China) into pUC57 cloning vector. Specific primers were designed according to the optimized gene on the basis of cloning each subunit individually. The synthetic gene was subcloned into pET28a, and the pET28a vector and pUC18 containing the chimeric gene was digested with *EcoRI* and *HindIII*. The linearized pET28a and the insert obtained from digestion of pUC18 were purified using the Bioneer Gel extraction kit (Bioneer, South korea). In total, 50 ng vector and 23 ng insert were mixed with 2 U of T4 DNA ligase (Fermentas, Lithuania) and T4 ligation buffer (300 mM Tris-HCl, 100 mM MgCl_2_, 100 mM DTT, and 10 mM ATP) in a 20-μl reaction mixture and incubated at 12^o^C overnight. The transformants were screened with restriction enzyme analysis and then confirmed by DNA sequencing. DNA fragments encoding *eltB* (312 bp) and *cstH* (438 bp) were ampliﬁed by PCR using synthetic gene as template and were cloned into pET28a and pET32a, respectively. 

***Expression and purification of recombinant proteins. ***The genes were expressed in *E*. *coli *BL21 (DE3). The transformants were grown in LB broth supplemented with 30 µg/ml concentration of kanamycin. The chimeric protein expression was induced with 1 mM IPTG upon OD_600_ of 0.5. The protein was analyzed by SDS-PAGE, and protein was purified by nickel-nitrilotriacetic acid (Qiagen, USA) resin under denaturing condition. The cell lysate was centrifuged twice at 14,000 ×g for 20 min, and supernatant was loaded in a nickel-nitrilotriacetic acid affinity column. The protein was eluted by pH gradient according to the manufacturer’s instructions. The eluted proteins were refolded in a step wise dialysis under descending concentrations of urea (from the 8 to 2 M) with a final dialysis in the absence of urea.

***Western blot. ***The purified protein extracts were separated on 12% SDS-PAGE and transferred to a nitrocellulose membrane (Sigma, USA). The membrane was blocked with 5% skimmed milk at 4°C overnight and then incubated with mice anti-His tag antibody (Abcam, UK) at 1:10,000 dilution in PBS-T (PBS + 0.05% Tween 20) at 37°C for 1 h. The proteins were detected using 3, 3'-diaminobenzidine as a substrate. 

***Animal immunization. ***A group of 10 female BALB/C mice were given four doses of each protein. First round immunization included 20 μg recombinant protein with complete Freund’s adjuvant injected subcutaneously and intraperitoneally. The second and third doses were given as boosters of 15 and 10 μg protein, respectively using incomplete Freund’s adjuvant. The last dose with 10 μg protein was administered without adjuvant. Blood samples were collected two weeks after each booster dose. The sera were maintained at -70°C. Purified chimeric protein (CS3) was used intramuscularly to immunize adult rabbits. A volume of 200,150,100, and 50 μg recombinant protein was homogenized in an equal amount of adjuvant injected on days 0, 14, 28, and 42, respectively. Blood samples were collected after each injection.

***Serum analysis. ***The level of antibodies against LTB, CS3, and CStH were determined by ELISA and compared with each other. Polystyrene 96-well plates (Thermo Scientific, USA) coated with 5 μg antigen in a coating buffer (64 mM Na_2_CO_3_, 136 mM NaHCO_3_, NaN_3_ pH 9.8) at 4°C overnight. The plates were washed with PBST after each step, and the non-specific sites were blocked with 5% skimmed milk powder in PBST at 37°C for 1 h. Immune and non-immune sera were serially diluted from 1:100 to 1:6400 in PBST, added to the ELISA plates and incubated at 37°C for 30 min. *Horseradish peroxidase* (*HRP*)*-*goat anti-mouse IgG (1:2,000 in PBST) (Abcam, UK) was added and then incubated at 37°C for 30 min. The development step was then performed using O-phenylenediaminedihydrochloride as a chromogen.

***GM1 ELISA. ***To assess the ability of recombinant anti-CS3 antibody in neutralizing native toxin and inhibiting its binding to GM1 receptor, the GM1-ELISA test was conducted. The heat-labile toxin was purified as described previously [20]. ELISA strips were coated by 0.5 μg GM1. After an overnight incubation at 4°C, skimmed milk in PBS (1:1, v/v) was added at 37°C for 1 h as a blocking step. Serum collected from immunized mice were serially diluted and treated with heat-labile toxin at 37°C for 1 h and then added to the wells. The plates were incubated at 37°C for 30 min and followed by washing and addition of 1:2000 dilution of HRP-goat anti-rabbit IgG (Sigma). The color development was performed using O-phenylenediaminedihydrochloride as a chromogen.

***Rabbit ileal loop assay. ***Bacteria were grown at 37°C for 72 h in brain heart infusion broth containing 2% casamino acids supplemented with polymyxin B (100 mg/ml). The cells were centrifuged at 12,000 ×g for 10 min, and the supernatants were filter sterilized to obtain cell-free supernatants. A white rabbit was kept at suitable condition and fasted for 24 h prior to use. Under local anesthesia, the small intestine was flushed with 10 ml PBS. A segment of small intestine was divided into three loops, each about 10 cm in length with 3 cm hiatuses*.* Intestinal loops were inoculated with cell-free supernatants from ETEC, ETEC + anti-LTB. The loops inoculated with PBS served as negative control. After injection of the loops, the abdomen was closed. The animal was sacrificed 18 h later by injection of pentobarbital into their veins. The loops were cut out, and the volume of fluid in each segment was measured. The lengths of the empty segments were determined, and the volumes per length ratios (ml/cm) were recorded [21]. Research was conducted in compliance with the Animal Welfare Act and regulations related to experiments involving animals.

***Caco-2 cell*** ***adherence inhibition assay. ***Overnight culture of ETEC in CFA broth was prepared and inoculated to fresh media. Cells were harvested at exponential phase, washed three times in PBS buffer and set the optical density to 0.5 at 600 nm. Caco-2 cells, which were grown in a culture flask containing RPMI with 10% FBS, were trypsinized and transferred to a sterile 6-well cell culture plate. In order to establish cellular monolayer, 8 × 10^4^ trypsinized cells were added to each well and incubated for 24-72 h. Bacterial cells (300 μl) pretreated with 40 μl immunized rabbit anti-sera were added to test wells. The plates were incubated at room temperature for 1.5 h to allow possible attachment. The cells were trypsinized and spread on LB agar plates. After 18 h incubation, the numbers of colonies in test and control plates were counted and the number of neutralized bacteria by antibody was calculated [22, 23].

***Statistical analysis. ***Statistical analyses were carried out by SPSS 12.0 and student *t*-test. Significant difference was demonstrated by *P*<0.05.

## RESULTS

**Fig.1:**
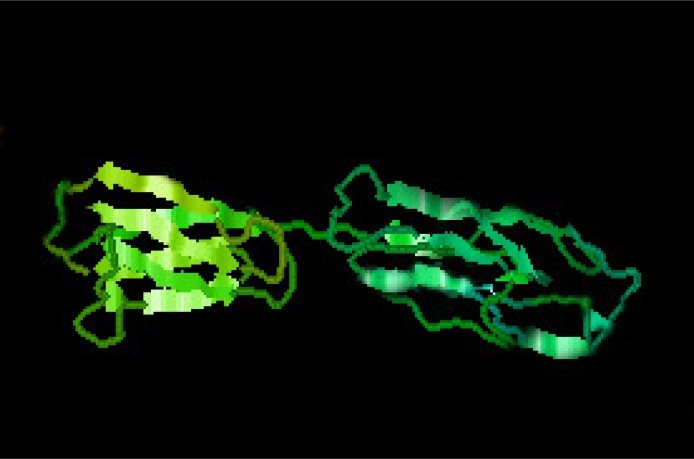
Modeled structure of chimeric protein by I-TASSER software

***Design and construction of chimeric gene. ***A synthetic chimeric sequence encoding the *cstH* and *eltB* genes was designed using *E. coli* codon bias. To optimize the synthetic gene, negative cis acting motifs and repeated sequences were avoided. Both the wild type and the synthetic chimera were analyzed for their codon bias and GC content. The overall GC content was improved from 38.96% to 48.75% upon codon optimization, which increased the overall stability of mRNA. ΔG of the best predicted structure was -147.5 kcal/mol. The nucleotides at the starting of the 5′ did not have a long stable hairpin or pseudoknot, whereas in the native mRNA, the ΔG was -112 kcal/mol. The chimeric gene showed a codon adaptation index of 0.96 compared to that of the wild-type gene, which was only 0.72. *Ab initio* modeling of the synthetic sequence was exploited to produce three dimensional models of the chimeric protein. The result of tertiary structure of the chimeric protein construction using I-TASSER showed a protein with two main domains linked together with a linker (Fig. 1).

**Fig. 2:**
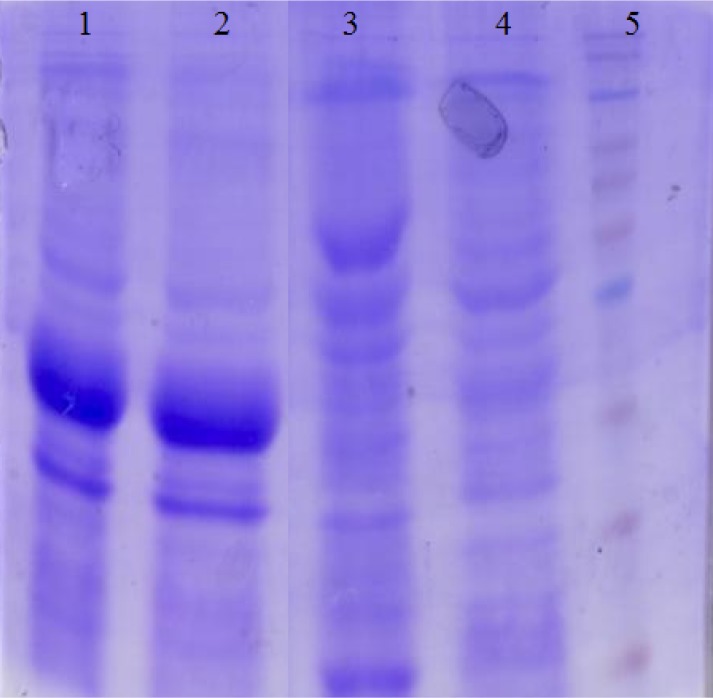
Expression of recombinant proteins analyzed by SDS-PAGE. Lanes 1 and 2, total proteins of *E. coli* BL21DE3/ pET28a-CstH:LTB induced with IPTG; lanes 3 and 4, un-induced *E. coli* BL21DE3/pET28a-CstH:LTB gene as control, and lane 5, protein molecular marker

**Fig. 3:**
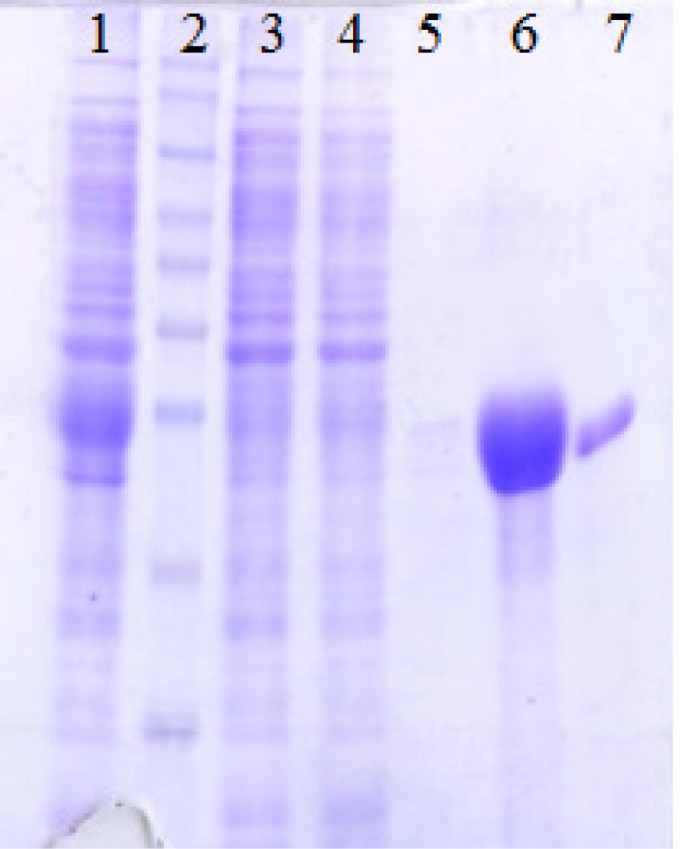
Purification of recombinant proteins with nickel-nitrilotriacetic acid column. Lane 1, expressed protein before purification; lane 2, protein molecular marker; lane 3, flow-through; lanes 4 and 5, wash column with C and D buffer (8M Urea, 20 mM NaH_2_PO_4_, 500 mM NaCl); lane 6, purified protein after elution with Elution buffer (8M Urea, 20 mM NaH_2_PO_4_, 500 mM NaCl), and lane 7, wash column MES (2-N-morpholine-ethanesulfonic acid) buffer

***Expression and purification of recombinant protein. ***The synthetic chimeric gene was expressed in *E. coli* (BL21DE3) with the N-terminal 6×-His tag and analyzed by SDS-PAGE (Fig. 2). The SDS-PAGE analysis showed the presence of a 33-KD recombinant chimeric protein. Purification of the recombinant chimeric protein was carried out under denaturing conditions, and SDS-PAGE analysis revealed the presence of the protein as a major band (Fig. 3). The expression of recombinant chimeric protein was confirmed by Western blotting using anti-His tag antibodies.

**Fig. 4:**
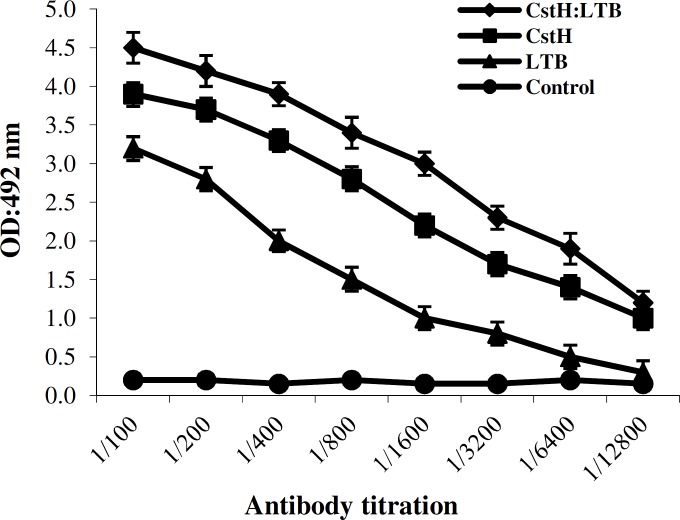
Evaluation of antibody production in animals receiving protein CstH:LTB, CstH, and LTB. Animals were injected with recombinant proteins using complete and incomplete Freund’s adjuvants. Immunizations were performed four times within eight weeks. Non-immunized mice sera were used as control (*P*<0.05).

**Fig. 5:**
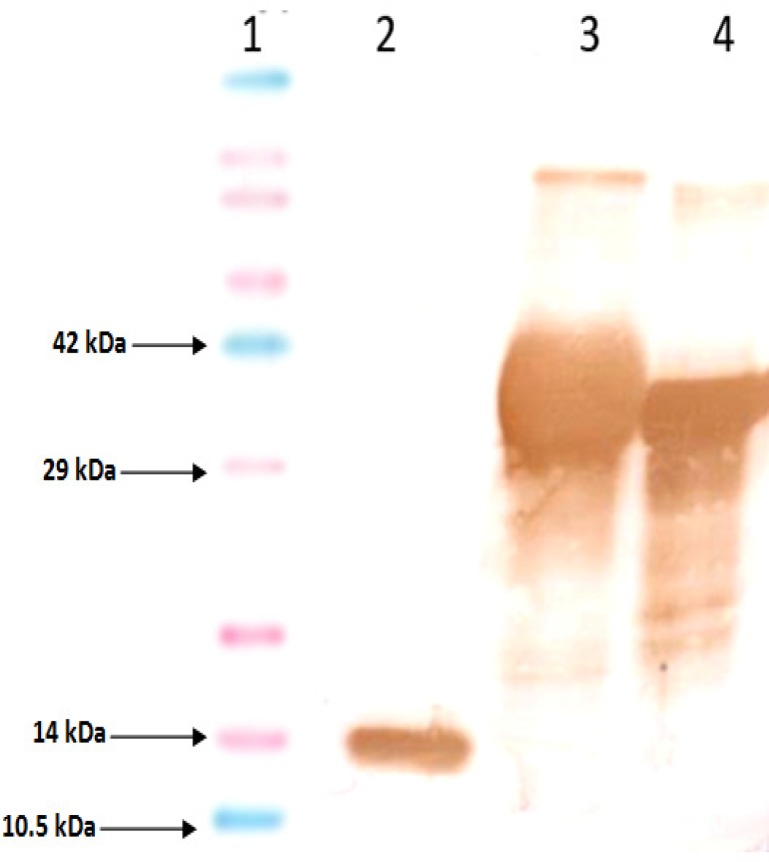
Western blot analysis of protein with CstH:LTB anti-serum. Lane 1, recombinant CstH:LTB protein reacted with CstH:LTB anti-serum; lane 2, CstH protein responded to CstH:LTB antibody; lane 3, recombinant LTB objected to Western blot with CstH:LTB anti-serum, and lane 4, protein molecular marker. Western blot analysis of protein with CstH:LTB anti-serum. lane 1, protein molecular marker; lane 2, recombinant LTB objected to Western blot with CstH:LTB anti-serum; lane 3, CstH protein responded to CstH:LTB antibody and Lane 4, recombinant CstH:LTB protein reacted with CstH:LTB anti-serum

***Immunization responses. ***The antigenicity of the recombinant proteins was determined by means of injecting CstH:LTB, CstH, and LTB into the mice and the rabbit subcutaneously and intraperitoneally. The humoral immune responses of the immunized mice were measured by ELISA technique. A high titer of antibody was produced in immunized animals. Remarkable titers of antibodies were noted after the second booster, which was further increased at subsequent booster doses (Fig. 4). The presence of LTB as an adjuvant resulted in elevated immune responses in comparison to the single antigen administration. The ability of anti-CstH:LTB antibody to recognize single proteins and also fusion one was verified by Western blot analysis (Fig. 5). 

**Fig. 6:**
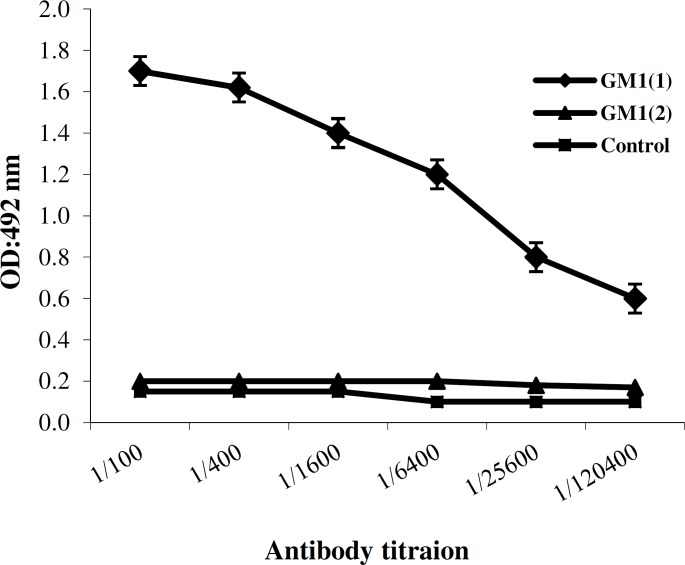
Determination of inhibition of the binding of heat labile to GM1 using GM1 ELISA assay. Each graph shows the mean OD ± SD in three independent experiments. GM1(1), GM1 binding to the native toxin as positive control; GM1(2), neutralized toxin by CstH:LTB anti-serum lost GM1 binding ability, and control, no GM1 coated in control strips

***Activity inhibition assay of toxin***. Biological activity and toxin neutralizing efficacy of the CstH:LTB-specific antibodies were tested by GM1-binding inhibition assay. Incubation of toxin with CstH:LTB antibody resulted in toxin neutralization and its prevention to bind to GM1 (Fig. 6). The sera from control mice or PBS did not show such effect. Induction of fluid accumulation by toxin was studied in rabbit ileal loops. The volume of secretion for each ileal loop was measured after excision. The fluid accumulation was not observed in rabbit ileal loops 18 h post infection with ETEC treated with the immunized serum. The volume per length ratios of ileal loops (ml/cm) of the rabbit injected with ETEC + anti-chimeric protein, ETEC, and PBS were recorded 0.81, 2.23, and 0.51, respectively. 

***Binding inhibition of ETEC to Caco-2 cells. ***ETEC cells pretreated with serum from the non-immunized rabbit were densely distributed on the Caco-2 cells, whereas ETEC-treated cells with rabbit anti-serum blocked their binding to Caco-2-cells. Immunized rabbit antibody inhibited 52% of bacterial cells to attach to Caco-2 cells compared to the ETEC cells treated with non-immunized rabbit serum.

## DISCUSSION

ETEC attachment to the enteric epithelial cells with the help of adhesions and induction of excessive fluid secretion by enterotoxins lead to diarrhea. Hence, obstructing adherence of bacteria and toxin activation through receptor binding are the main strategies for prevention and control of ETEC disease [15]. Trial vaccines including whole-cell *E*.* coli *strains, which express a toxin and an adhesion antigen or joining both antigens as subunit vaccines, would confer anti-adhesion and anti-toxin immunity to the receiving hosts [18]. Administration of CS3 antigen combined with heat-labile toxoid in a purified form results in antibody development against both CS3 and LT antigens in human volunteers [24]. In this study, we fused* cstH *and* eltB* coding sequences with a suitable (EAAAK)4 linker, and (EAAAK)4 sequences were introduced among different domains for more flexibility and efficient separation. Our successful experience of using four repeated EAAAK sequences in chimeric gene has shown that it could lead to logically acceptable results [19, 22]. 

The fusion has been synthesized because of codon optimization applied according to *E. coli* codon bias. The *cstH* subunit was selected in chimera construction because of being the major fimbrial subunit, and it seems to be the most important antigen in CS3 operon to elicit antibody. Likewise, LTB selection depended on its non-toxic nature and also its immune stimulatory and adjuvant activity, which triggers B-cell differentiation and antibody production [25]. Several studies have been approved LTB as a potent adjuvant in chemical or genetic integration or in simultaneous administration with other antigens [11, 26-29]. Consequently, heat labile can function both as an antigen and an adjuvant [26]. In consistence with these references [27-30], our findings also indicated that the chimer protein consisting LTB and CstH had better immune responses than that of CstH alone.

Anti-His tag antibody utilization in Western blotting approved CstH:LTB integrity. Additional approval was added by the demonstration of the antigenic determinants of both CstH and LTB using anti-CstH:LTB antibody.

Recombinant strains expressing CstH:LTB were capable of inducing anti-CS3 and anti-heat-labile immunity, and immunization was particularly revealed more effective antibody responses than immunization with each antigen alone [10]. The main purpose in using booster doses after firs injection was to accomplish excellent immune responses and activate the B cell immune cascade by decreasing antigen doses [27]. After immunization with the chimeric protein, animals developed high titers of antibodies capable of reducing fimbrial attachment to Caco-2 cells and also neutralized toxin as shown in GM1 ELISA and ileal loop assay.

In conclusion, the recombinant fusion protein induced anti-adhesion antibodies against ETEC. The protein could also neutralize native toxin and inhibit its binding to GM1 receptor. The designated chimer is protective against ETEC. 
